# Extra-Limites

**Published:** 1834-04-01

**Authors:** 


### EXTRA-LIMITES.

### Dr. Wise's Reply to Mr. Arnott.

TO THE EDITOR OF THE MEDICO-CHIRURGICAL REVIEW.

SIR,

I trust you will be good enough to allow the following statement to appear in an early Number of your valuable Journal, having reference to a Letter addressed by Mr. Arnott to Dr. Johnson, and published in your October Number for 1832.

Mr. Arnott's anger appears to have been particularly directed against Dr. Johnson, for having permitted certain remarks, authenticated by the name of a gentleman in India, to appear in the Medico-Chirurgical Review, animadverting on the character of a surgeon to a London hospital, without their having been first submitted to that individual for perusal, with a view, I conclude, of his adding or erasing such parts as might suit his peculiar purpose. The satisfactory and triumphant manner in which you have, in the October Number, vindicated the line of conduct pursued on this occasion must, I think, have carried conviction to the heart of every unprejudiced man, and proved that you were perfectly justified in publishing the communication in question.

Mr. Arnott very complacently states, that the period which had elapsed between the publication of his Essay and the appearance of my Statement, ought to have prevented your inserting the latter document; or, in other words, that the distance at which an individual resides, or his opportunities of seeing new publications, should be the test which ought to guide the Editor of a journal in rejecting or inserting communications. Had you adopted this rule for your guide, I should never have been able to expose Mr. Arnott's proceedings.

His Essay I have not yet seen; and the first intimation I received of his publication was from your review, and that many months after it had reached this country, as medical works, I assure you, are not very easily procurable in the interior of Bengal. I then learned, for the first time, that Mr. Arnott had, a very few months after I left England, presented an Essay to the Medico-Chirurgical Society on the subject of Phlebitis. That could not have been written without a long and patient examination of various medical works, and must have been in hand at the very time when I was candidly communicating to him my opinions on the nature of this disease, which Mr. Arnott well knew I was making my particular study, and on which he confidentially further knew it was my intention to publish, had I not been obliged to delay the undertaking, in consequence of my immediate departure for India.

Annoyed as I very naturally was on discovering this want of candour on the part of Mr. Arnott, it was not until the year 1831, that I had fully ascertained the extent to which he had appropriated to himself the information communicated by me on this subject—at a time, too, when he had led me to suppose that he was engaged in very different pursuits. In that year I had occasion to look over my papers, with a view to publish the two Essays that I forwarded to you; and, in so doing, became convinced of the necessity of pointing out the cause of the striking resemblance between Mr. Arnott's remarks and my own, and deemed it essential to my own character to send you, with the Essays, the memorandum or note pointing out the extraordinary conduct of Mr. Arnott. I now trust that I shall be able to prove, to the satisfaction of every one who has taken an interest in this question, that my former statement contained nothing but what is borne out by undeniable facts. Passing over the publication of the very *same three cases*, which of course, with Mr. Lawrence's permission, Mr. Arnott had an equal right to with myself, I solicit your attention for a few moments, while I compare Mr. Arnott's results, as given in your Review, with the few observations which I added to these very *same* three cases which were printed by me in 1827, and of which I beg to enclose a printed copy for your own inspection, and that of any gentleman who may take an interest in this discussion.

Mr. Arnott commences his paper as follows:—"A degree of doubt seems still to prevail as to the cause of the alarming constitutional affection frequently attendant on inflammation of the veins, and much obscurity unquestionably exists, with regard to the origin of those abscesses, and inflammation in distant parts which sometimes occurs after injuries. An attempt to remove the one, and to dispel a portion of the other, may not therefore be considered as altogether unworthy of notice." This is the categorical intention of Mr. Arnott's Essay; and it bears a peculiar and certainly curious resemblance to the following few remarks added to the cases printed by me. Other consequences of phlebitis less prominent were not stated, although detailed at length in my two Essays. "As the symptoms of internal inflammation advanced, these in the vein proceeded more slowly. * * * These constitutional symptoms were severe inflammatory fever, and inflammation of a serous membrane, which is illustrated by the above cases, and by a considerable number of other fatal cases of the disease which I have witnessed, and is still further supported by experiments which I made upon animals. * * * As the disease proceeded pus was discharged from the wound in the vein, or from abscesses in the cellular tissue. The inflammatory fever was followed by symptoms of depression which generally resembled very much those of the epidemic (typhus) fever of this country. * * * The examination after death proved, that by the local effects of the bleeding or other exciting causes, an inflammation of the vein and surrounding cellular substance occurred, in consequence of which either from the structure or function of the part inflamed, inflammation of other and distant parts followed, attended with much danger to life. Such effects may be compared to fever, accompanied with inflammation of the mucous membrane of the intestines, or syphilis, with the affection of the throat, &c."

The local disease was thus stated as not being progressive with the constitutional effects, which are fever taking on the typhoid form, and inflammation of the cellular, serous, and synovial membrane, at a distance from the primary local disease, with distant purulent deposits.—*(See Essays.)*

The reviewer of Mr. Arnott's Essay (p. 36) says, that throughout the Essay, and in the latter part of it especially, he (Mr. Arnott) evinces remarkable talent for what is termed "framing" a theory. We admire the ingenuity, but deplore the instability of the workmanship. Mr. Arnott laboured under disadvantages in explaining his ideas; he wished to give information, as far as he could do so without compromising himself —this is seen in his explanation of the termination of inflammation in the veins which he says "has *escaped* the *notice* of those who have *previously treated* of inflammation of veins." His explanation accords with what I have stated in my Essays, (p. 77, et seq.); but, he was obliged to leave out the peculiar changes in the blood, which is so remarkable a consequence, from being aware I had published a note in the Medical and Physical Journal for the year 1827, (a copy of which I have added to the communication, (No. 2.) This note was published at the *suggestion of Mr. Arnott*. A knowledge of the facts therein stated, explaining the facility with which he happily imagined the last paragraph of his Essay, which he delivers with all the circumstances of an oracle-finis que coronat opus.—"As the object of these remarks was to point out the relation between the primary and secondary affections in phlebitis, and to establish the introduction of pus or other inflammatory secretion from the surface of the vein into the circulation as the cause of the latter, I do not regard the matter so deposited to be actually that which has been brought into the circulation from the inflamed vein or veins, (although no reason is given why the pus in the vein should not enter the circulation.) The disease of the eye in which pus is not deposited, and the affection of the joints, exclusive of other considerations, clearly prove that the question is no longer one of a translation of matter merely, but one which involves the difficult subject of the pathology of the blood, especially *the share* which *diseased changes in this fluid* have in the production of those phenomena which we are in the habit of comprehending under the term of inflammation."—(P. 123.)

After perusing the above, what are we to think of Mr. Arnott's declaration, (Med. Chir. Rev. October, 1832, p. 504,) "that he has not derived any cases, materials, facts, or opinions, from Dr. Wise."! What are we to think of his statement that he never saw a dissection or drawing (*See* Essays) or knows what Dr. Wise's opinions may be on the subject of phlebitis. Several gentlemen could testify, that in pointing out to Mr. Arnott the cases of erysipelas then in St. Bartholomew's Hospital, when going my rounds as House-Surgeon, accompanied almost daily by my *friend* Mr. Arnott, I confided to him what I had formerly witnessed of the course and consequences of phlebitis, explained what I considered the course of the constitutional effects of the disease as they were developed in the *very three cases*—and I believe the *only original cases* published in Mr. Arnott's Essay, which occurred at that time in St. Bartholomew's Hospital—and to which I particularly directed his attention, as being extremely interesting in themselves, and as affording proofs of the justness of the conclusions at which I had previously arrived. Hence is explained Mr. Arnott's acknowledgment (p. 2.) "that his attention was more particularly *called* to the subject by *some circumstances* which marked the course and termination of the '*same*' three fatal cases of the inflammation of the veins after venesection, which I (Mr. Arnott) had an *opportunity* of *observing*." Lastly, what are we to think of Mr. Arnott's conduct, when I state that he saw my remarks on those three cases, which were drawn out at his suggestion, and printed for the London Medical and Physical Journal,* and Mr. Arnott himself corrected one if not two of the proof-sheets ! ! !

The above facts will readily explain why Mr. Arnott, instead of answering my assertions found it easier to attack Dr. Johnson, for giving insertion to my communication. I only trust that I have been as successful in vindicating myself as he was in repelling the insinuation of improper motives ; and if I have not been so fortunate, I hope the candid reader will attribute it to a desire to condense my proofs, and entreat him, if at all interested in the subject, to pause in drawing an unfavourable conclusion as to my motives for appearing before the public, until he has read and compared my Essays (imperfect as I am aware they are) with the communication of Mr. Arnott to the Medico-Chirurgical Society.

I have many apologies to offer, Mr. Editor, for having written at such length ; which I trust will be attributed to the natural desire of explaining my motives for having addressed you before in vindication of my rights, and now in reply to the aspersions of Mr. Arnott. I am, Sir,

Your most obedient Servant,

*Hooghly, Bengal, 29th April,* 1833. THOS. S. WISE, M.D.

P.S. I must add that Dr. Johnson is only known to me by his high and well merited reputation ; nor am I aware that I am personally known to any individual writer in the Medico-Chirurgical Review.

*** The cases alluded to, printed in 1827, by G. Black, Bartholomew Close, have been transmitted to the Editor, and are in his possession.

---

* I dare say the then learned Editor, Dr. M'Leod may recollect the circumstance, from his having been inconvenienced by their being withdrawn ; which, however, he politely permitted for reasons which it is unnecessary now to mention.

